# RT001 in Progressive Supranuclear Palsy—Clinical and In-Vitro Observations

**DOI:** 10.3390/antiox10071021

**Published:** 2021-06-25

**Authors:** Plamena R. Angelova, Kristin M. Andruska, Mark G. Midei, Mario Barilani, Paldeep Atwal, Oliver Tucher, Peter Milner, Frederic Heerinckx, Mikhail S. Shchepinov

**Affiliations:** 1 Department of Clinical and Movement Neurosciences, UCL Queen Square Institute of Neurology, Queen Square, London WC1N 3BG, UK; p.stroh@ucl.ac.uk; 2 California Movement Disorders Center, Los Gatos, CA 95032, USA; kristinandruska@cmdcneuro.com; 3 Retrotope, Los Altos, CA 94022, USA; paldeep@retrotope.com (P.A.); oliver@retrotope.com (O.T.); peter.milner@retrotope.com (P.M.); frederic@retrotope.com (F.H.); 4 Laboratory of Regenerative Medicine–Cell Factory, Department of Transfusion Medicine and Hematology, Fondazione IRCCS Cà Granda Ospedale Maggiore Policlinico, via F. Sforza 35, 20122 Milano, Italy; m.barilani@gmail.com

**Keywords:** PSP, lipid peroxidation, RT001, PUFA, mesenchymal stem cells, deuteration

## Abstract

Progressive supranuclear palsy (PSP) is a progressive movement disorder associated with lipid peroxidation and intracerebral accumulation of tau. RT001 is a deuterium reinforced isotopologue of linoleic acid that prevents lipid peroxidation (LPO) through the kinetic isotope effect. The effects of RT001 pre-treatment on various oxidative and bioenergetic parameters were evaluated in mesenchymal stem cells (MSC) derived from patients with PSP compared to controls. In parallel, 3 patients with PSP were treated with RT001 and followed clinically. MSCs derived from PSP patients had a significantly higher rate of LPO (161.8 ± 8.2% of control; *p* < 0.001). A 72-h incubation with RT001 restored the PSP MSCs to normal levels. Mitochondrial reactive oxygen species (ROS) overproduction in PSP-MSCs significantly decreased the level of GSH compared to control MSCs (to 56% and 47% of control; *p* < 0.05). Incubation with RT001 significantly increased level of GSH in PSP MSCs. The level of mitochondrial DNA in the cells was significantly lower in PSP-MSCs (67.5%), compared to control MSCs. Changes in mitochondrial membrane potential, size, and shape were also observed. Three subjects with possible or probable PSP were treated with RT001 for a mean duration of 26 months. The slope of the PSPRS changed from the historical decline of 0.91 points/month to a mean decline of 0.16 points/month (+/−0.23 SEM). The UPDRS slope changed from an expected increase of 0.95 points/month to an average increase in score of 0.28 points/month (+/−0.41 SEM). MSCs derived from patients with PSP have elevated basal levels of LPO, ROS, and mitochondrial dysfunction. These findings are reversed after incubation with RT001. In PSP patients, the progression of disease may be reduced by treatment with RT001.

## 1. Introduction

Progressive supranuclear palsy (PSP), or Steele–Richardson–Olszewski syndrome, is a sporadic, progressive neurodegenerative disease characterized by ocular motor dysfunction, postural instability, akinesia, and cognitive dysfunction. Freezing of gait, levodopa resistance, behavioral changes, and aphasia are often seen [1]. Symptoms typically begin after age 60 but can begin earlier. The exact cause of PSP is unknown. PSP is often misdiagnosed as Parkinson’s disease, Alzheimer’s disease, corticobasal syndrome, and other neurodegenerative disorders [2,3,4,5]. At present, no effective therapies exist [6].

Neuropathological examination of the post-mortem PSP brain reveals intracerebral aggregation of the microtubule-associated protein tau in neurofibrillary tangles throughout the brain, most prominently in the brainstem, deep cerebellar nuclei, and basal ganglia [7]. LPO byproducts such as toxic aldehydes are selectively associated with neurofibrillary tangles in the PSP patients [8]. A regionally specific increase in LPO has been observed [9], and other reports have demonstrated defects in oxidative phosphorylation in muscle mitochondria from PSP patients [10]. Cerebrospinal fluid increases in superoxide dismutase and glutathione conjugated with 4-hydroxynonenal further support the association of LPO with PSP [11]. Evidence of damage to proteins rendered inactive by toxic aldehydes and insoluble neurofibrillary tangles in the brain of PSP patients firmly establish the role of LPO within the causal pathway of PSP. These observations indicate a potential therapeutic role for a drug that can downregulate isoprostanes, reactive aldehyde generation, and LPO in such patients.

RT001 is a deuterated isotopologue of linoleic acid that makes membrane PUFAs resistant to LPO. A strong protective effect against LPO is seen when deuterated PUFAs replace non-deuterated PUFAs in liposomal lipid bilayers at levels exceeding 20% [12]. Treatment with RT001 has shown early signs of efficacy in patients with Friedreich’s ataxia, a disorder of intracellular free-iron imbalance that initiates LPO, resulting in increased oxidative stress and mitochondrial dysfunction [13]. RT001 is also being developed in other diseases associated with lipid peroxidation, including infantile neuroaxonal dystrophy and amyotrophic lateral sclerosis.

## 2. Materials and Methods

The current study evaluated the effect of RT001 on various oxidative and bioenergetic parameters in MSCs derived from patients with PSP. We also report on the results of expanded access use of RT001 in 3 patients with PSP, each of whom was treated for over 27 months.

### 2.1. In Vitro Methods

MSC preparations from bone marrow (BM) were obtained from control subjects and from the PSP subjects following previously described protocols [14,15,16,17,18]. Passage 4–6 MSCs were used for all experiments. BM collection from PSP patients was authorized by the Ethics Committee of Fondazione IRCCS Ca’ Granda Ospedale Maggiore Policlinico (Italy), the National Health Institute (Istituto Superiore di Sanità), and approved by the Italian Medicines Agency (Agenzia Italiana del Farmaco, AIFA). The trial is registered at ClinicalTrials.gov (NCT01824121). All BM donors gave their written informed consent.

### 2.2. Live Cell Imaging

Lipid peroxidation was measured using C11-BODIPY 581/591 (2 μM; Molecular probes) at 488 and 543 nm excitation and emission measured from 505 to 550 nm and 560 nm. For assessments of glutathione levels, 50 μM monochlorobimane (MCB) (Molecular Probes, Invitrogen) was used and images of the MCB-GSH fluorescence were acquired using a Zeiss 710 CLSM with excitation at 405 nm and emission at 435–485 nm. Mitochondrial ROS generation rate was assessed using MitoTracker^®^ Red CM-H2XRos (Thermo Fisher Scientific, Waltham, MA, USA) which accumulates in mitochondria upon oxidation. The fluorescence measurement was obtained by excitation with 561 nm laser and emission was detected above 580 nm. Mitochondrial membrane potential (ΔΨm) was assessed using 25 nM tetramethylrhodamine methyl ester (TMRM, Thermo Fisher Scientific) at 560 nm excitation and fluorescence was measured above 580 nm. Z-stack images were collected and the fluorescence intensity of TMRM was analyzed using Zen 3.4 (Blue Edition) software (Zeiss).

In vitro RT001 effects on lipid peroxidation, mitochondrial function, glutathione, mitochondrial membrane potential, mitochondrial number, and mitochondrial structure of 3 lines of PSP-MSC were compared to the effects on MSCs derived from 2 healthy control age-matched subjects. H2-LA and RT001 were added to cultures as described previously [17] and the data pooled for each of the conditions applied.

### 2.3. Clinical Methods

Patients with PSP participated in this study at the Parkinson’s Institute, Mountain View, CA and at the California Movement Disorders Center, Los Gatos, CA. Ethics board approval was obtained, and all participants gave written informed consent.

Participants met the Movement Disorders Society clinical diagnostic criteria for possible or probable PSP [4] with Richardson Syndrome. Participants underwent baseline assessment using the 28-item Progressive Supranuclear Palsy Rating Scale (PSPRS) [19] and the Unified Parkinson’s Disease Rating Scale (UPDRS) [20,21]. They were then treated with RT001 (2.88 g BID; 5.76 g total daily dose) and observed for disease progression. Subject 2 increased the dose (2.88 g TID; 8.64 g total daily dose) after the first year of treatment. During the treatment period, scores in the two rating scales were determined every 3 months. Pharmacokinetic (PK) sampling was performed at month 3. These analytes included plasma and RBC membrane levels of D2-linoleic acid (D2-LA) and its centrally active metabolite D2-arachidonic acid (D2-AA).

## 3. Results

### 3.1. In Vitro Results

**Figure 1:**
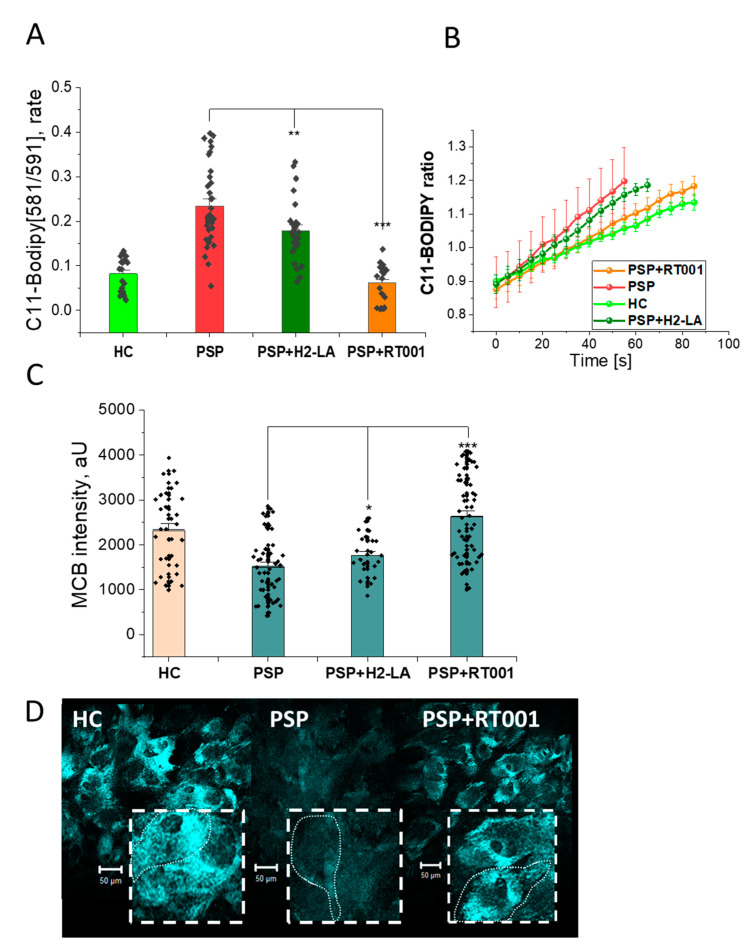
Effects of RT001 on the oxidative status of MSCs derived from healthy controls (HC) and patients with PSP (PSP). (**Panel A**) Bar chart quantification of the efficacy of RT001 on the rate of lipid peroxidation using C11-Bodipy (PSP alone vs. PSP + RT001, *p* < 0.0001). (**Panel B**) Representative time course traces of lipid peroxidation in MSCs derived from HC (light green), and PSP (red), PSP treated with RT001 (orange), and PSP treated with H2-LA (dark green), respectively. (**Panel C**) Measurements of monochlorobimane (MCB) fluorescence intensity as an indicator of glutathione (GSH) levels (PSP alone vs. PSP + RT001, *p* < 0.0001). (**Panel D**) Representative images showing MCB (GSH) fluorescence intensity for HC, PSP, and PSP + RT001. MCB intensity is reduced in PSP compared to HC, but is restored after incubation with RT001. The coarse dash lines approximate the cell borders of an individual MSC (fine dash line). Data are represented as mean ± SEM. Total number of cells per well *n* = 10–50 from 3–6 culturing wells. All experiments were repeated 2–3 times (N, independent culturing conditions). * *p* < 0.05, ** *p* < 0.001, *** *p* < 0.0001.

Using the LPO-specific probe BODIPY C11, PSP MSCs had a significantly higher rate of LPO compared to controls. After a 72-h incubation of the cell lines with RT001, PSP MSCs returned to normal levels, while PSP MSCs incubated with non-deuterated linoleic acid ester (H2-LA) remained elevated (Figure 1, Panel A). The time course of lipid peroxidation for the various MSCs and treatments is displayed in Figure 1, Panel B.

Glutathione levels were measured using MCB. The MCB intensity was reduced in PSP MSCs compared to HC. After incubation with RT001, glutathione levels were restored to HC levels, while glutathione levels remained low in PSP MSCs after incubation with H2-LA Figure 1, Panel C. Representative images of these cells are shown in Figure 1, Panel D.

**Figure 2:**
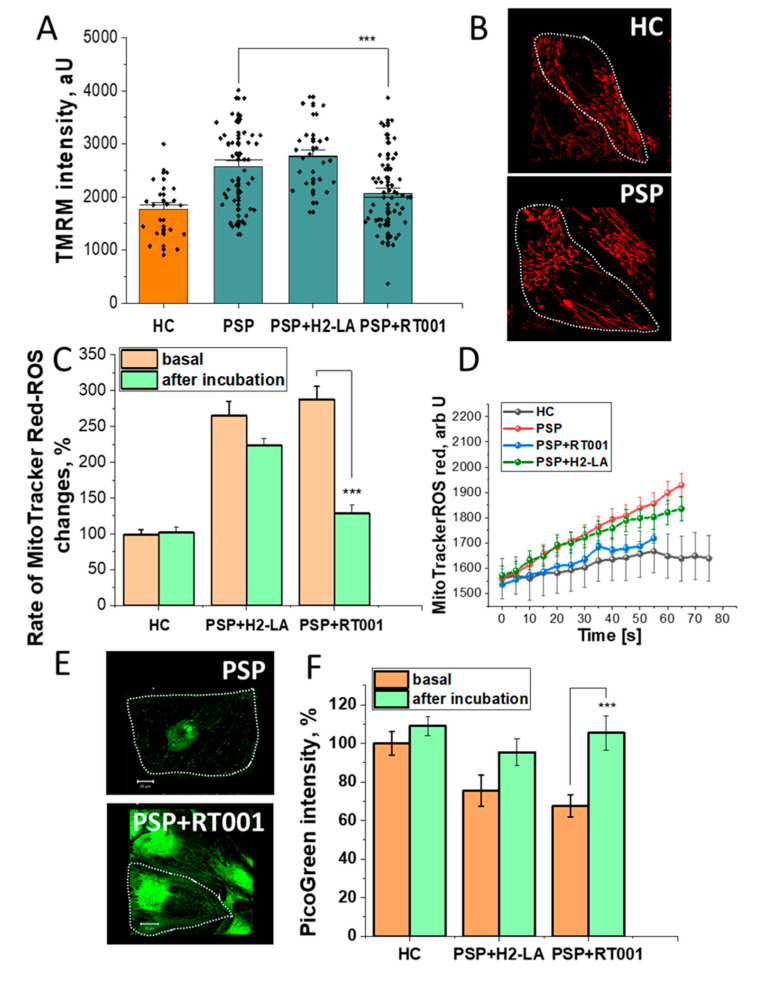
Protective effects of RT001 on mitochondrial function in PSP-MSCs. (**Panel A**) Histogram demonstrating the mitochondrial membrane potential (Δψm) measured using the fluorescence intensity of TMRM (tetramethylrhodamine). Δψm was increased in PSP MSCs compared to HC. Δψm was reduced after incubation with RT001, but not after incubation with H2-LA (*p* = 0.0009). (**Panel B**) Representative images depicting the mitochondrial shape, distribution, and fluorescence intensity at baseline for HC and PSP MSCs (fine dash line represents the approximate cell border of a MSC). (**Panel C**) Quantitative histogram of MitoTrackerCM-H2Xros fluorescence intensity shows baseline elevations in ROS in the PSP MSCs were reduced to near normal levels after incubation with RT001 (*p* < 0.0001), but not after incubation with H2-LA (*p* = 0.0801). (**Panel D**) MitoTrackerCM-H2Xros fluorescence over time for HC, baseline PSP, PSP + RT001, and PSP + H2-LA. Baseline fluorescence elevations for the PSP MSCs over HC MSCs were restored to near normal after RT001, but not after H2-LA incubation. (**Panel E**) Representative images of the mitochondrial DNA content of PSP MSCs at baseline (top panel). (**Panel F**) Quantification bar chart of the PicoGreen Intensity as a measure of mitochondrial DNA content. Baseline (orange columns) reductions in mitochondrial DNA were seen in the PSP MSCs (middle and right histograms). Incubation with H2-LA (middle histogram) resulted in a small increase in mitochondrial DNA (middle histogram, green column; *p* = 0.0689), while RT001 restored PSP MSCs to normal levels (right histogram, green column; *p* = 0.0010). Data are represented as mean ± SEM. Total number of cells per well n = 10–50 from 3–6 culturing wells. All experiments were repeated 2 – 3 times (N, independent culturing conditions). * *p* < 0.05, ** *p* < 0.001, *** *p* < 0.0001.

The fluorescence intensity of TMRM was increased in the PSP MSCs relative to HC MSCs, indicating an increase in the Δψm. This increase remained elevated when the cells were incubated with H2-LA, but Δψm normalized after RT001 (Figure 2, Panels A and B). The changes seen in the Δψm were also seen in the fluorescence of MitoTrackerCM-H2Xros. Fluorescence intensity of MitoTrackerCM-H2Xros for the PSP MSCs was increased more than 2.5 times HC at baseline, indicating an increase in mitochondrial ROS. Incubation with H2-LA reduced mitochondrial ROS generation slightly, but RT001 reduced mitochondrial ROS back to near normal levels (Figure 2, Panels C and D). Fluorescence intensity with Pico Green exhibited an inverse correlation with the other studies. PicoGreen fluorescence was decreased at baseline for the PSP MSCs relative to HC, indicating a reduced amount of mitochondrial DNA. After incubation with H2-LA, fluorescence increased slightly, but was far more pronounced for the RT001 incubated cells. In addition to increased mitochondrial DNA amount, the structure and number of mitochondria were also increased by RT001 treatment (Figure 2, Panels E and F).

### 3.2. Clinical Results

Baseline demographic information for the three subjects is displayed in Table 1. Two subjects with a diagnosis of probable PSP-RS were enrolled and one subject had possible PSP-RS. Within the first year of the study, the subject with possible PSP-RS developed signs that were consistent with probable PSP-RS. Although each participant in the expanded access trial had undergone MRI in the past, the stored images did not permit area calculation of the midbrain-pontine ratio. The studies did confirm the absence of other confounding pathologies, however.

**Figure 3:**
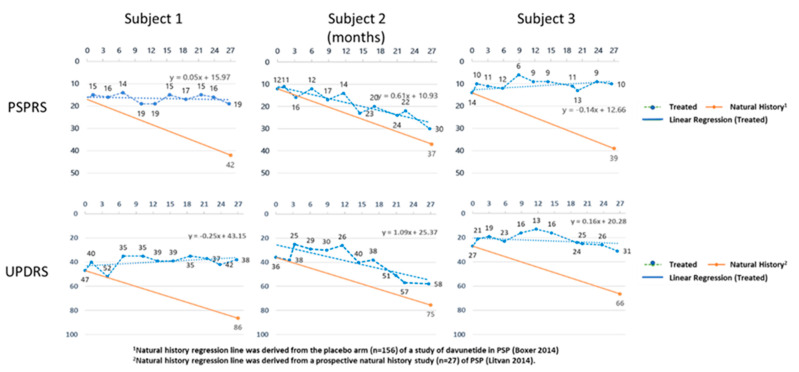
The PSPRS and UPDRS scores for the three subjects are shown over time. The orange line indicates the expected change in scores based on historical control subjects obtained from the placebo arm of a clinical trial in PSP patients for the PSPRS [22], and in a natural history study of PSP for the UPDRS [23].

In all of the treated subjects, we saw a slowing of the rate of decline in both scales. In fact, for one of the subjects, the PSPRS improved slightly (Subject 3), and in another, the UPDRS improved slightly (Subject 1). The linear regression slopes of the PSPRS and UPDRS scores for the three subjects were plotted against those obtained from disease progression predicted by previous longitudinal studies of untreated PSP patients. Figure 3 shows the slope of the PSPRS changed from the historical decline of 0.91 points/month to a mean of decline of 0.16 points/month (+/−0.23 SEM). The UPDRS slope changed from an expected increase of 0.95 points/month to an average increase in score of 0.28 points/month (+/−0.41 SEM).

### 3.3. Pharmacokinetics

Mean plasma and RBC membrane levels of drug were 21% and 19% of total linoleic acid. Levels of di-deuterated arachidonic acid in both plasma and RBC also increased, indicating normal enzymatic processing of the stabilized LA into stabilized AA.

### 3.4. Clinical Safety

Overall, RT001 was well tolerated. Subject 2 experienced the only serious adverse event during the expanded access trials. At the time of the event, the subject was a 77-year-old male who had been taking RT001 for 18 months. On the day before admission, amantadine had been prescribed. After the subject took the second dose of amantadine, he developed lower extremity weakness and a fall that required hospitalization for 3 days. The initial evaluation with a non-contrast head CT scan was unremarkable. Also complicating his clinical situation was a lactic acidosis while taking metformin. Amantadine and metformin were discontinued and symptoms resolved within 24 h. The discharge diagnosis was presumed to be a transient ischemic attack or an adverse drug reaction to either amantadine or metformin. The investigator determined that the event was unrelated to RT001 and treatment was never interrupted. Symptoms have not recurred despite continued RT001 treatment.

## 4. Discussion

RT001 reduced LPO and mitochondrial ROS production and improved other measures of mitochondrial health in MSCs derived from patients with PSP. Oral RT001 was well tolerated in three subjects with PSP over a minimum treatment period of 24 months, and this treatment was associated with a stabilization in the rate of decline in functional rating scales over time.

The pivotal role for increased ROS and LPO in the pathophysiology of PSP has been identified previously [18]. Because of its down-regulation of LPO, RT001 affords a novel yet specific approach to preventing these harmful effects on lipids in mitochondrial and other membranes in PSP. Improvement in these in vitro parameters were seen with RT001 pre-treatment, leading to improvement in mitochondrial number, function, and structure. Treatment of PSP with RT001 may be a reasonable therapy to interrupt the causal pathway leading to mitochondrial dysfunction, tau accumulation, and cell death.

In PSP, misfolded and aggregated tau incorporates into plasma and mitochondrial membranes, causing depolarization and flux through various ion channels. The resulting cellular and mitochondrial calcium overload activates cytosolic and mitochondrial ROS production, calcium-induced Caspase-3 activation, and cell death cascades [24,25]. Incorporation of RT001 and RT001-derived D2-AA into cell membranes should reduce LPO and PUFA degradation compounds such as toxic isoprostanes and bifunctional aldehydes and should ultimately reduce the neurodegenerative consequences of tau accumulation.

In addition to tau, other intrinsically disordered proteins like alpha-synuclein and beta-amyloid are characterized by the formation of aggregates that have similar membrane perturbation capacity. Restoration of membrane oxidative status with RT001 prevents the acute aggregate-membrane interaction, calcium dysregulation, and cell death in human IPS-derived neurons with triplication of alpha-synuclein [26]. Thus, RT001 has the potential to be effective in other types of neurodegenerative diseases in which protein misfolding and lipid peroxidation are pathophysiologic [27].

Slowing in the rate of decline of functional rating scales is a common method for evaluating the success or futility of a drug intervention in neurodegenerative disorders. Although the improvements that we saw may indicate disease reversal, these were very small changes and are probably within the limits of variability. They are encouraging signs, however, and will be best evaluated in the randomized, double-blind clinical trial that is currently underway.

The interpretations of the clinical results reported here are subject to the inherent limitations of an open-label study without concurrent placebo controls. However, previous studies have suggested the absence of a significant placebo effect in PSP clinical trials [28]. Because this initial clinical experience included only three subjects treated for 24 months, the current study is further limited by the small sample size and by the duration of treatment. Further exploration of the effects of RT001 in PSP is warranted in a randomized, placebo-controlled trial of appropriate size and duration.

## 5. Conclusions

In summary, in vitro studies of MSCs derived from PSP patients demonstrate increased rates of LPO and mitochondrial dysfunction that can be reversed with RT001 pre-treatment. Expanded access treatment of PSP patients with RT001 slows the rate of PSP progression. RT001 represents a potential therapy for PSP patients that should be studied in randomized, controlled clinical trials.

## Figures and Tables

**Table 1:** Demographic and baseline PSP characteristics for the three subjects at the onset of treatment.

Characteristic	Subject Number		
	1	2	3
Age (years)	66	73	74
Sex	Male	Male	Female
Pre-treatment symptom duration (years)	6	3	2
Diagnosis	Probable PSP-RS	Possible PSP-RS	Probable PSP-RS
Baseline PSPRS	17	12	13
Baseline UPDRS	44	36	21

## Data Availability

Data is contained within the article. Additional detail regarding the data presented in this study are available on request from the corresponding author. The data are not publicly available due to privacy restrictions.
